# Drug Weaponry to Fight Against SARS-CoV-2

**DOI:** 10.3389/fmolb.2020.00204

**Published:** 2020-08-25

**Authors:** Elena Cabezón, Ignacio Arechaga

**Affiliations:** Instituto de Biomedicina y Biotecnología de Cantabria (IBBTEC), Universidad de Cantabria-CSIC, Santander, Spain

**Keywords:** coronavirus, SARS-CoV-2, Covid-19, spike, drugs, ACE-2, polymorphisms

## Abstract

The current outbreak of SARS-CoV-2 virus has caused a large increase in mortality and morbidity associated with respiratory diseases. Huge efforts are currently ongoing to develop a vaccine against this virus. However, alternative approaches could be considered in the fight against this disease. Among other strategies, structural-based drug design could be an effective approach to generate specific molecules against SARS-CoV-2, thus reducing viral burden in infected patients. Here, in addition to this structural approach, we also revise several therapeutic strategies to fight against this viral threat. Furthermore, we report ACE-2 genetic polymorphic variants affecting residues involved in close contacts with SARS-CoV-2 that might be associated to different infection risks. These analyses could provide valuable information to predict the course of the disease.

## Introduction

Severe acute respiratory syndrome coronavirus (SARS-CoV) and Middle East respiratory syndrome coronavirus (MERS-CoV) have been responsible for major outbreaks, causing the death of hundreds of patients (Ksiazek et al., 2003; Kuiken et al., 2003; Rota et al., 2003; Anderson et al., 2004; Assiri et al., 2013; De Wit et al., 2016; Song et al., 2019). The current pandemic spread of SARS-CoV-2 virus, responsible for the coronavirus disease 2019 (Covid-19), has driven health systems around the world to the edge of collapse. Covid-19 has a lower lethality rate than SARS and MERS, which emerged in 2002 and 2012, respectively. However, and precisely because of its lower fatality, SARS-CoV-2 virus has widely spread around the world. The rate of infection by this virus is superior to other common respiratory viruses, such as influenza, with a larger basic reproductive number (R_*o*_), and a higher case fatality rate (CFR). Nonetheless, it is not possible to know exactly the infective and fatality rate numbers, as it is very likely that there are many undetected, infected people. What is certain is that the world is in a state of emergency in the fight against this disease. Several initiatives are currently on the way with special focus on development of vaccines to protect populations against this infection.

Vaccines are the main tool to prevent the spread of the infection in the long term. Unfortunately, they have little impact in patients already infected with the virus. Therefore, therapeutic drugs should be found to treat infected patients at hospitals. Several drugs are under consideration to deal with the disease, although most of them have not been specifically designed to target this virus. In the battery of available therapeutic drugs we can find antivirals, such as remdesivir, lopinavir, ritonavir, ribavirin, and fapiravir. Molecules affecting interactions of SARS-CoV-2 with different cell partners are also being used in clinical setups, albeit with no clear benefits. Some examples are the anti-paludic chloroquine (Colson et al., 2020; Gao et al., 2020) and its derivative hydroxy-chloroquine (Liu et al., 2020) combined with azithromycin (Gautret et al., 2020), a broad-spectrum antibiotic. However, several studies question the effectiveness of chloroquine, and articles on this matter have been retracted (Mehra et al., 2020a, b). Recently, the WHO has recommended against the use of high doses of chloroquine for treatment of Covid-19.

A recent high-throughput mass spectrometry analysis found that SARS-CoV-2 interacts with multiple cellular systems (DNA replication, host translation machinery, RNA processing and replication, innate immune system pathways, vesicle trafficking, lipid modification, nuclear transport, mitochondria, etc.) (Gordon et al., 2020). Therefore, instead of trying to develop new drugs, some trials are underway to test compounds already approved for human use, in the hope of finding one that might affect those interactions. This strategy would try to stop the cell from making new viral particles out of viral RNA, thus preventing virus translation (Gordon et al., 2020). However, most of the efforts are focused on the viral spike protein that SARS-CoV-2 uses to infect cells, in a strategy that seeks to disrupt the virus entry. SARS-CoV-2 binds to the angiotensin conferring enzyme receptor (ACE-2) with high affinity, via the spike protein (Hoffmann et al., 2020; Wrapp et al., 2020). Therefore, the use of antibodies to neutralize the binding of the virus to ACE-2 has also been considered in the treatment of the disease (Kruse, 2020), as well as the use of peptides that mimic the interaction between the virus and ACE-2. Thus, molecules that could prevent the binding of the virus to ACE-2 would be of great interest to treat this disease. Here, we describe in detail the interactions between SAR-CoV-2 and ACE-2, and the main therapeutic strategies that are in consideration for the treatment of Covid-19 to inhibit virus entry or virus translation, but with a particular emphasis on the viral spike protein, since most of the efforts are focused on this target.

## Coronavirus Structure

**FIGURE 1:**
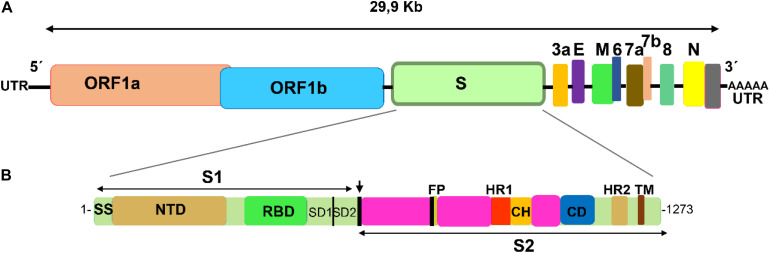
Schematic representation of SARS-CoV-2 genome and spike functional domains. **(A)** SARS-CoV-2 single stranded positive RNA contains two large ORF genes that encode 16 non-structural proteins, and four genes that encode four essential structural proteins: the spike (S), the envelope (E), the membrane (M), and the nucleocapsid (N). In addition, the genome contains a number of accessory genes (3a, 6, 7a, 7b, and 8). **(B)** The spike gene encodes two spike subunits, S1 and S2. The cleavage site between S1 and S2 is indicated with an arrow. The S1 subunit contains a domain (RBD, receptor binding domain) close to the C-terminus, which recognizes and binds the angiotensin-II conferring enzyme receptor (ACE-2). Fusion and entry of the virus into the receptor cell is mediated by the S2 subunit, which contains a fusion protein (FP) and two heptad repeat motifs (HR1 and HR2). For comparison, image follows the schematic representation for SARS-CoV in Song et al. (2019).

Coronaviridae is a family comprising several viral species (Lai et al., 2007; Adams and Carstens, 2012), seven of which are able to infect human cells (the *alphacoronavirus* HCoV-229E, HCoV-NL63, and the *betacoronavirus* HCoV-OC43, HCoV-HKU1, SARS-CoV, MERS-CoV, and SARS-CoV-2) (Kin et al., 2015; Su et al., 2016). These pleomorphic membrane-enveloped viruses consist of a positive sense RNA molecule and four essential structural proteins: M (the most abundant glycoprotein in the membrane), E (an envelope small membrane protein), N (a nucleocapsid protein), and S (the spike protein, which is also a membrane glycoprotein) (Figure 1). The spike glycoproteins (S) form homotrimers that decorate the viruses (Du et al., 2009; Kirchdoerfer et al., 2016; Walls et al., 2016; Wrapp et al., 2020; Yan et al., 2020). The spike protein is essential for binding the receptor and for its entry into the infected cell (Gallagher and Buchmeier, 2001; Bosch et al., 2003; Li et al., 2006). During the course of infection the S protein is cleaved by host proteases in two fragments, the S1 and S2 subunits, which remain non-covalently bound in the prefusion conformation (Bosch et al., 2003; Belouzard et al., 2009; Millet and Whittaker, 2014; Walls et al., 2020). The S1 subunit contains a Receptor Binding Domain (RBD) which interacts with the cell receptor (Babcock et al., 2004; Li, 2015), whereas the S2 subunit acts in the fusion and entry into the cell (Walls et al., 2020). The S2 subunit is a multidomain protein consisting of a cytoplasmic domain, a transmembrane span, a fusion peptide, and two heptad repeats (HR1 and HR2) (Bosch et al., 2004; Liu et al., 2004; Xia et al., 2020b). These two heptad repeats oligomerize into a six-helix bundle fusion core, which is essential for viral integrity and infectivity. There has been much effort in developing peptides based on HR1 and HR2 structures to prevent infection by these viruses, mainly for MERS-CoV and HCoV-229E, with promising results (Gao et al., 2013; Lu et al., 2014; Sun et al., 2017; Xia et al., 2018). However, the entry pathway of MERS-CoV into the cell, mediated by the DPP4 receptor (Meyerholz et al., 2016), is different from that used by both SARS viruses, SARS-CoV and SARS-CoV-2, which bind to the ACE-2 receptor. Therefore, these peptides may be helpless in the current outbreak.

## Interactions of the Spike Protein With the ACE-2 Receptor

**FIGURE 2:**
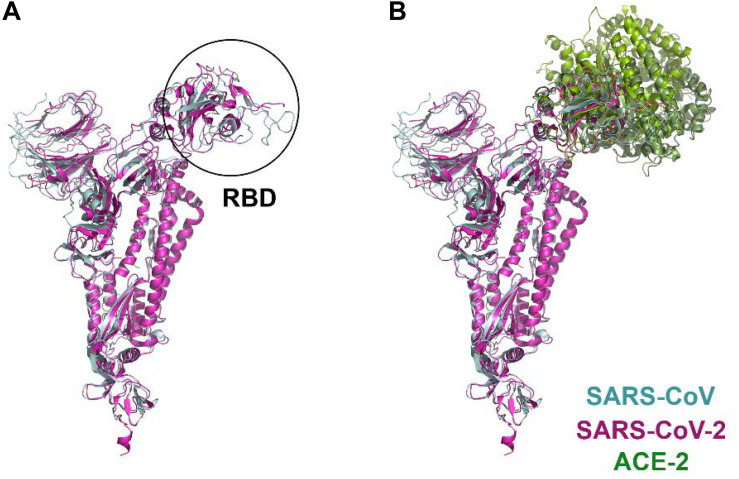
Structural comparison of SARS-CoV and SARS-CoV-2 spike proteins in complex with the ACE-2 receptor. **(A)** protomer superposition of SARS-CoV (*blue*) and SARS-CoV-2 (*pink*). **(B)** structures of the complex formed by the RBD of SARS-CoV (*blue*) and ACE-2 (*light green*) (PDB id: 2ajf; Li et al., 2005), and the RBD of SARS-CoV-2 (*pink*) and ACE-2 (*dark green*) (PDB id: 6vw1, Shang et al., 2020a; PDB id: 6m17, Yan et al., 2020; PDB id: 6lzg, Wang Q. et al., 2020), respectively, have been aligned and superimposed using UCSF Chimera (Pettersen et al., 2004).

The spike S1 glycoprotein of SARS like viruses interacts very strongly with ACE-2, a protein receptor involved in the maturation of angiotensin, an essential peptide in vascular homeostasis (Donoghue et al., 2000; Crackower et al., 2002). ACE-2 receptor is a membrane protein, consisting of an N-terminal domain, named PD, and a C-terminal Collectrin-like domain (CLD) (Zhang et al., 2001). Structures of the ACE-2 PD domain in complex with SARS-CoV receptor domain (RBD) have been published (Li et al., 2005, PDB id: 2AJF). Recently, the atomic structure of the spike glycoprotein of SARS-CoV-2 has been determined (Wrapp et al., 2020, PDB id: 6VSB; Walls et al., 2020, PDB id: 6VXX and 6VYB; Supplementary Figure S1). Moreover, the structure of the complex formed between the RBD of the viral spike and ACE-2 receptor has also been solved (Shang et al., 2020b, PDB id: 6VW1; Yan et al., 2020, PDB id: 6M17 and 6M18; Wang Q. et al., 2020, PDB id: 6LZG; Figure 2). Despite the high degree of homology shared by the spike glycoproteins of SARS-CoV and SARS-CoV-2 viruses, monoclonal antibodies directed against the SARS-CoV of 2002/3 virus were not effective against the new SARS, revealing important differences between the two spike viral proteins (Wrapp et al., 2020).

**FIGURE 3:**
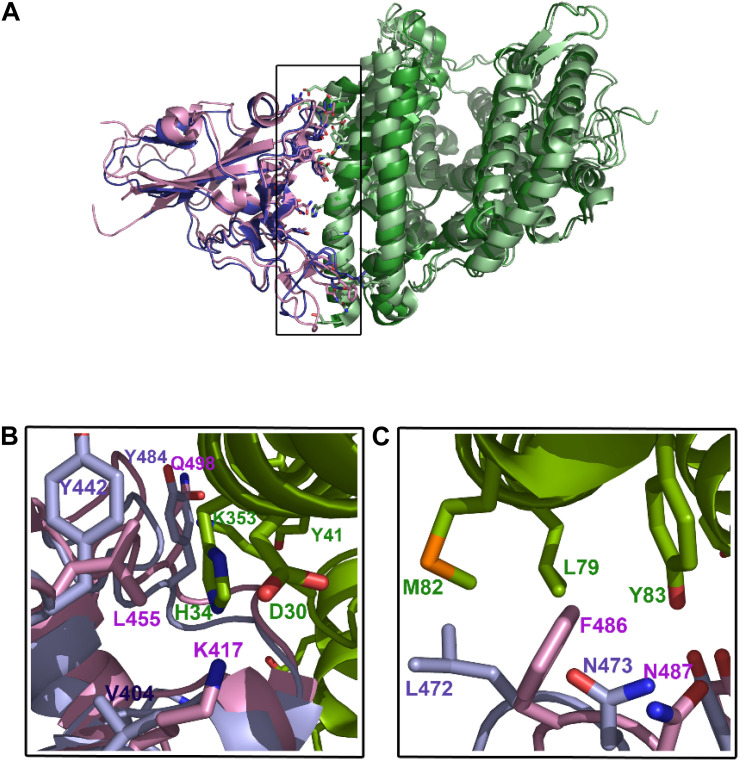
Interacting region between SARS-CoV-2 and ACE2. **(A)** Structures of the RBDs of both SARS-CoV (*slate blue*) and SARS-CoV-2 (*pink*) in complex with ACE-2 receptor (*green*). **(B)** amino-acid residues of SARS-CoV-2 RBD domain (*pink*) interacting with the N-terminus and C-end of the α1-helix of ACE-2 receptor (*green*). Equivalent positions in SARS-CoV (2002/3) are shown (*light blue*). **(C)**, the main differences between both spike viral proteins are the substitution of Val404 for Lys417, which favors the polar interaction with Asp30 of ACE-2, and the substitution of Leu472 for Phe486, which enables the formation of Van der Waals bonds with Met82 and hydrophobic interactions with Tyr83.

Sequence comparison between the SARS-CoV and SARS-CoV-2 spike proteins revealed 76% identity (Supplementary Figure S2). The main sequence variations are precisely in the RBD domain involved in ACE-2 interaction. Although the interface between ACE-2 and SARS-CoV-2 (Yan et al., 2020) is similar to that of ACE-2 with SARS-CoV (Li et al., 2005), a close inspection of this area reveals some important differences (Wang Y. et al., 2020). Among these, substitutions of Asn^479^ and Thr^487^ in SARS-CoV for Gln^493^ and Asn^501^ in SARS-CoV-2, respectively, seem to be particularly relevant, as they are key residues in the interaction with the ACE-2 receptor. Up to six other modifications in SARS-CoV-2 are present in this region (Arg^426^→Asn^439^, Tyr^442^→Leu^455^, Leu^443^→Phe^456^, Phe^460^→Tyr^473^, Leu^472^→Phe^486^, and Tyr^484^→Gln^498^; SARS-CoV→SARS-CoV-2 mutations) (Supplementary Figure S3). In addition to these substitutions, it is worth noting the change of Val^404^ in SARS-CoV for Lys^417^ in SARS-CoV-2 (Figure 3). The introduction of a positive charge in this position contributes to the formation of a salt bridge with residue Asp^30^ in ACE-2 (Yan et al., 2020). Likewise, the change of Leu^472^ for Phe^486^ seems to favor the formation of strong Van der Waals interactions with Met^82^ in the receptor (Yan et al., 2020). Other residues in this region of interaction between SARS-CoV and SARS-CoV-2 with ACE-2 are well conserved. Overall, these modifications seem to increase the stability of the complex formed between the RBD of SARS-CoV-2 and ACE-2 receptor, which is in agrement with the high affinity (K_*d*_ = 15 nM) reported for the binding between both proteins (Wrapp et al., 2020). This extraordinary affinity could explain the high rates of infection observed in Covid-19.

A recent analysis of 5,349 SARS-CoV-2 genomes (Phelan et al., 2020) has found numerous SNPs (Single Nucleotide Polymorphisms), although at low frequencies. Only three mutations are more prevalent, two mutations in the nucleocapsid protein and one in the spike protein (D614G). This D614G mutation has been suggested to increase the morbility and mortality of the disease (Eaaswarkhanth et al., 2020; Korber et al., 2020). In any case, despite this mutation, the spike protein is relatively stable, which is good news for the development of vaccines. Nonetheless, a close inspection of further mutations is paramount in order to prevent failures in the design of a vaccine.

Finally, another point to consider is the glycosylation of the spike protein, since it could facilitate the hiding of specific epitopes, which in turn would neutralize antibody recognition by the immune system. Glycosylation has been described for a number of coronaviruses (Walls et al., 2019; Yang et al., 2020). A recent site-specific mass spectrometry analysis (Watanabe et al., 2020) has enabled mapping of the glycosylation sites on the viral SARS-CoV-2 spike. These viral glycans were found to be different from typical host glycans. They are mainly oligomannosides and complex fucoside-derived sugars localized in 22 different sites on the spike protein (Watanabe et al., 2020). It is not clear yet how this glycosylation pattern affects the interaction with the ACE-2 receptor but this type of study could provide a framework to develop future glycoprotein-based vaccines.

## ACE-2 Genetic Variants That Might Be Associated With a Different Risk for Covid-19 Outcome

Recently, it has been shown that ACE-2 expression levels may be critical for the susceptibility and outcome of Covid-19 (Cao Y. et al., 2020). In a systematic analysis of ACE-2 coding-region variants and expression quantitative trait loci (eQTL) variants, the authors found that East Asian populations have increased allele frequencies in the eQTL variants associated with high ACE-2 expression, suggesting an enlarged susceptibility to SARS-CoV and SARS-CoV-2 infection. However, no clear evidence of resistant mutations for coronavirus spike-protein binding was detected (ChinaMAP and 1KGP databases). In this context, a genetic analysis for coding variants affecting ACE-2 expression is of particular interest, since ACE2 gene polymorphisms and ACE2 mRNA expression might influence the disease outcome (Devaux et al., 2020).

Genetic variants in the ACE-2 receptor affecting the interaction with the spike protein might be associated with a different risk of SARS-CoV-2 infection. The ACE-2 gene is associated with 6634 variant alleles (Ensembl GRCh38.p13), and three of them are missense variants that affect three essential residues involved in close contacts between the RBD of the spike protein and the ACE-2 receptor (Table 1). The missense variants E37K and E329G would affect H-bonds and essential polar contacts with residues of the spike protein, whereas M82I variant could have a bearing on the van der Waals interactions established with a leucine or a phenylalanine in SARS-CoV and SARS-CoV-2 viruses, respectively. In particular, molecular modelling of E329G variant showed noticeable variations in the interactions with the viral spike protein (Hussain et al., 2020). Although these are very low-frequency missense variants, found in the gnomAD database (MAF < 0.01), it might be interesting to investigate whether they are associated with a lower risk of infection, corresponding to that percentage of the population that shows very weak symptoms or are practically asymptomatic.

**TABLE 1:** ACE-2 missense variants affecting residues involved in SARS-CoV and SARS-CoV-2 spike-protein binding.

ACE-2 variant	Polymorphism	Allele frequency*	Codons	SARS-CoV interaction	SARS-CoV-2 interaction
E37K	rs146676783	9.1 × 10^–5^	GAA/AAA	Y491	Y505
M82I	rs766996587	1.4 × 10^–4^	ATG/ATT/ATA	L472	F484
E329G	rs143936283	9.1 × 10^–5^	GAA/GGA	R426	N439

*Source: Genome aggregation database (gnomAD database).

The ACE-2 receptor forms a dimer, which can fluctuate between two conformations, “open” and “closed” (Towler et al., 2004; Yan et al., 2020). However, when ACE-2 is bound to the RBD of the spike protein, only the closed state is present, which suggests that residues stabilizing the dimer interface might be also essential for virus infection. Table 2 shows genetic variants in the ACE-2 receptor affecting essential residues involved in dimerization. Missense variants affecting this interface might also be associated to a lower risk of SARS-CoV-2 infection.

**TABLE 2:** ACE-2 missense variants affecting residues involved in ACE-2 dimerization.

ACE-2 variant	polymorphism	Allele frequency*	Codons	ACE-2 dimer interaction
N638S	rs183135788	1.8 × 10^–4^	AAT/AGT	R652, Q653
S709R	rs1052746182	2.8 × 10^–5^	AGC/CGC	R716
R710H	rs370187012	4.6 × 10^–5^	CGT/CAT	N636
R710C	rs901495523	9.2 × 10^–5^	CGT/TGT	N636
R716H	rs200540199	1.4 × 10^–4^	CGT/CAT	S709,D713
R716C	rs144869363	2.8 × 10^–5^	CGT/TGT	S709,D713

*Source: Genome aggregation database (gnomAD database).

In addition, recent studies on the biology of viral infection also indicate that there might be a sex predisposition to develop Covid-19, with men more prone to being infected (Cai, 2020; Ciaglia et al., 2020; Guan et al., 2020). It is worth noting that the ACE-2 gene is located on the X chromosome and, therefore, allele variants associated to an increased risk of infection would affect women and men differently. As ACE-2 genetic variants are at different frequencies in the general population, it might be worthwhile investigating whether they confer a genetic predisposition for a different risk of infection. This knowledge would be of great interest to improve disease management, but additional investigation will be required to confirm such a putative association.

## Therapeutic Strategies

### Immunotherapy

Antibodies that recognize the epitope domain of previous SARS-CoV have been reported (Sui et al., 2004; Ter Meulen et al., 2004; Qiu et al., 2005; van den Brink et al., 2005), as well as monoclonal antibodies (m396 and S230.15) that neutralize SARS-CoV/ACE2 interaction (Zhu et al., 2007). Some of these antibodies (m396) bind to the RBD of the viral spike with high affinity (Prabakaran et al., 2006), thus preventing the binding of the virus to ACE-2. Unfortunately, none of these antibodies were able to recognize SARS-CoV-2 (Wrapp et al., 2020). Therefore, production of specific monoclonal antibodies against SARS-CoV-2 should be a priority. A cryptic epitope highly conserved in the RBD of SARS-CoV and SARS-CoV-2 has been identified (Yuan et al., 2020) and antibodies (CR3022) directed against it have been shown to bind with high affinity *in vitro* (Tian et al., 2020). Generation of antibodies by phage display could also be an alternative strategy. In that sense, antibodies already developed against HR1 and HR2 domains of the S2 spike of SARS-CoV (Elshabrawy et al., 2012) and other viruses (Shin et al., 2019) could provide leads to generate specific antibodies against SARS-CoV-2. However, more research is needed before it can be translated to clinical practice. Other alternative approaches, such as purifying polyclonal antibodies from animals, do not look promising. Passive immunotherapy with antibodies from plasma of already infected people might be an alternative therapy (Walker and Burton, 2018). Unfortunately, the variability in plasma samples and viral cargo of previously infected people makes this approach less precise and reproducible. Another clinical trial for the treatment of Covid-19 is based on Tocilizumab, a humanized monoclonal antibody commercialized by Roche to treat rheumatoid arthritis, which is being used in Covid-19 infected patients with promising results (Fu et al., 2020; Guo et al., 2020).

Novel strategies that rely on targeting the viral receptor protein in the cell surface, preventing the binding and entry of the virus inside the cell, could be more promising. For instance, fusions of immunoglobulin Fc with a soluble fraction of ACE-2 has been proposed to prevent the binding of the virus to the receptor (Kruse, 2020). As additional benefit, this therapy would supplement ACE-2 levels during infection, and it would boost the immune system to generate lasting immunity (Kruse, 2020).

Another strategy has been to generate fusion proteins containing the extracellular domain of human ACE2 and the Fc region of the human immunoglobulin IgG1 to neutralize SARS-CoV or SARS-CoV-2 spike proteins *in vitro* (Lei et al., 2020). As the authors state, these fusion proteins have potential applications in the diagnosis, prophylaxis, and treatment of SARS-CoV-2.

### Peptides That Prevent Interactions Between ACE-2 and SARS-CoV-2

In addition to the generation of antibodies, treatment with antiviral peptides could provide an alternative route of therapy to avoid virus entry. In contrast to small inhibiting molecules, peptides have the advantage of disrupting relatively large protein-protein interfaces. Antiviral peptides were designed against MERS-CoV (Gao et al., 2013; Lu et al., 2014; Sun et al., 2017) and also against SARS-CoV virus in order to disrupt the interaction between the spike protein and the ACE-2 receptor (Hu et al., 2005) or affect the interface of the spike trimer (Zheng et al., 2005). Peptides selected against the S2 spike subunit (Yuan et al., 2004; Xia et al., 2018) and peptides including short sequences of the RBD of SARS-CoV were also proposed (Struck et al., 2012). Recently, and based on the pan-coronavirus virus fusion inhibitor EK1, developed for SARS-CoV and MERS-CoV (Xia et al., 2019), other lipopeptides, such as EK1C4, have been produced (Xia et al., 2020a), which could be effective in the treatment of Covid-19.

Close inspection of the amino acid substitutions in the interacting region between SARS-CoV-2 spike protein and ACE-2 receptor provides a framework to generate a library of antigenic peptides. These peptides should block the interaction of the virus with target cells. To this respect, some reports have been recently published in which peptides specifically designed to bind SARS-CoV-2 spike protein have been generated (Zhang et al., 2020). These peptides should be able to disrupt SARS-CoV-2 RBD-ACE2 interface. One of these peptides, named SBP1, is a 23-mer molecule including amino acid residues 21–43 from ACE-2 α1-helix (IEEQAKTFLDKFNHEAEDLFYQS) (Supplementary Figure S4). This peptide is able to bind the RBD of SARS-CoV-2 at nanomolar levels (Zhang et al., 2020), but it is unknown if this peptide is tolerated by the immune system.

Interestingly, another short region in ACE-2 involved in closed contacts with the spike can be appreciated (residues 325–354) (Supplementary Figure S3), which could be a lead to develop other effective peptides. The affinity or stability of such a putative peptide has not been assayed, but it might be worth testing it, alone or in combination with other peptides.

The use of a soluble fraction of ACE-2, instead of small peptides, to snatch viral particles has been suggested (Batlle et al., 2020). Such soluble ACE-2 recombinant protein has already been used in the treatment of angiotensin II-dependent hypertension (Wysocki et al., 2010), but its potential use in the treatment of Covid-19 is unknown.

### Specific Proteases

Another therapeutic alternative is the use of specific proteases. The spike protein of previous SARS-CoV is cleaved by a host transmembrane Type II serine protease TMPRSS2 (Glowacka et al., 2011; Reinke et al., 2017). This cleavage results in the activation of the spike protein, which, in turn, facilitates the entry of the virus into the cell. Thus, development of therapeutic drugs targeting this protein could also open an alternative route in the treatment of patients (Simmons et al., 2011). In this regard, some serine protease inhibitors targeting TMPRSS2 were proposed (Kawase et al., 2012; Zhou et al., 2015). Recently, it has been shown that SARS-CoV-2 entry into the cells is also mediated not only by ACE-2 but also by TMPRSS2 (Hoffmann et al., 2020). Moreover, this entry is blocked by camostate mesylate, a TMPRSS2 serine protease inhibitor, which makes this molecule a potential drug to be used in clinical trials (Hoffmann et al., 2020). Endosomal cysteine proteases, like cathepsin B and L (CatB/L), might also be inhibited with compounds such as E64 (Hoffmann et al., 2020). Interestingly, these authors also found that sera from patients infected with SARS-CoV neutralized SARS-CoV-2 entry into the cell.

*In silico* analyses of putative protease SARS-CoV-2 inhibitors are being used to identify potential therapeutic compounds (Pant et al., 2020). Among the potential inhibitors found in this analysis, there are some already described antivirals, such as lopinavir and ritonavir, and other FDA approved drugs, like cobicistat and darunavir.

### Furin-Like Enzymes

Binding and release of viral particles is activated by specific cellular proprotein convertases, such as furin, trypsin, and cathepsin-L (Seidah and Prat, 2012; Millet and Whittaker, 2015; Izaguirre, 2019). Therefore, development of drugs that target the furin-like process mechanisms constitutes another avenue of research. For instance, teicoplanin, an antibiotic used to treat *Staphylococcus* infection, has been found to inhibit cathepsin-L in several coronavirus, including SARS-CoV-2 (Baron et al., 2020). Based on the crystal structure of furin, several inhibitors, like 2,5-dideoxystreptamine-mediated inhibitor, were previously described and tested in clinical trials against SARS-CoV (Dahms et al., 2017). This drug is now a promising candidate for Covid-19 treatment. Some cell furin-like proteases are able to target the spike protein of several coronavirus species, including SARS-CoV-2 (Coutard et al., 2020). Interestingly, the furin cleavage site in SARS-CoV-2 site differs from that present in other coronavirus (Coutard et al., 2020). However, as furin-like molecules are involved in multiple metabolic pathways, any drug targeting these molecules might have serious adverse effects.

### Antivirals

Various essential stages in the viral life cycle, such as RNA synthesis, are susceptible to be targeted by drugs. In that sense, several antivirals are being used in the treatment of patients affected by Covid-19 (Table 3). Among them we can find adenosine nucleotide analogs, such as remdesivir, a broad spectrum antiviral agent with activity against a number of different virus, including Ebola virus (Siegel et al., 2017; Tchesnokov et al., 2019) and pathogenic coronavirus SARS-CoV and MERS-CoV (De Wit et al., 2016). Remdesivir inhibits viral RNA polymerase, thus reducing viral load. Remdesivir has been approved by the FDA to be used in the treatment of patients infected with SARS-CoV-2, since compassionate treatment with remdesivir has been reported to improve the outcome in several patients (Grein et al., 2020). Combined treatment of remdesivir with chloroquine has also been suggested to inhibit SARS-CoV-2 (Wang Y. et al., 2020). Other tested antivirals such as lopinavir, an HIV-1 protease inhibitor which, combined with ritonavir, was used against SARS and MERS (Chu et al., 2004; Chan et al., 2015) has also been tried with Covid-19 patients (Cao B. et al., 2020). Ribavirin, a nucleoside analog developed against influenza and hepatitis C virus (Feld and Hoofnagle, 2005; Te et al., 2007), has also being used in animal models infected with MERS in combination with interferons (IFNs) (Al-Tawfiq et al., 2014), but its use against respiratory diseases could not be advisable, as it reduces hemoglobin concentration (Sheahan et al., 2020). A combination therapy with lopinavir, ritonavir, ribavirin, and interferon a has been proposed as treatment of MERS-CoV infections (Kim et al., 2015). Favipiravir, an RNA dependent RNA polymerase (RdRp) inhibitor (Furuta et al., 2013), has been used against Ebola and Marburg virus (Bixler et al., 2018), influenza (Goldhill et al., 2018), and many other RNA viruses.

**TABLE 3:**
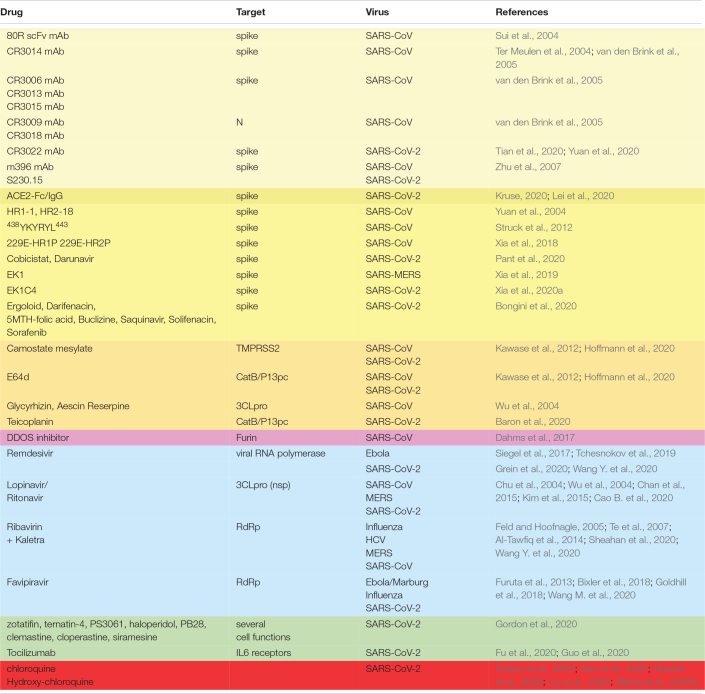
Drugs proposed for the treatment of SARS-CoV-2 infected patients.

*Compounds affecting the entry of the virus into human cells include antibodies (*light yellow*), small peptides and drugs disrupting binding to the ACE-2 receptor (*dark yellow*), and specific proteases (*salmon*). Strategies affecting virus translation involve furin-like enzymes (*pink*), antivirals (*light blue*), and other inhibitors affecting essential proteins and cell functions (*light green*). Chloroquine and derivatives (red), although initially proposed as potential drugs to treat SARS-CoV-2, have been lately dismissed.*

However, none of these antivirals were specifically designed against SARS-CoV-2. Therefore, development of specific antivirals against SARS-CoV-2 is a foremost objective.

### Other Potential Therapeutic Treatments

An alternative strategy could be the use of small interfering RNA (siRNA) to target directly the viral RNA (Qureshi et al., 2018). However, administration of these molecules to the patients presents practical problems. A similar approach was tried against Ebola and, despite of initial success in preclinical animal tests (Thi et al., 2015), it failed in clinical trials.

Another approach could be to target viral assembly. The formation of the S spike trimer might not be a fast and direct process, so there is a chance to block the quaternary structure of the spike (Bongini et al., 2020). Analysis of protein-protein interfaces of the spike has led to the prediction of ligands (Ergoloid, Darifenacin, 5-methyl-tetrahydrofolic acid, Buclizine, Saquinavir, Solifenacin, Sorafenib, tetrahydrofolic acid) that potentially could affect this process (Bongini et al., 2020).

In addition to the generation of specific peptides directed to neutralize SARS-CoV-2 spike or the action of the serine protease TMPRSS2 already mentioned in the previous section, other targets, such as the viral non-structural proteins (*nsp*) or the accessory proteins (envelope, nucleocapsid, and membrane) could be considered. Coronavirus *nsp*s are relatively well conserved (Totura and Bavari, 2019; Kim et al., 2020). Among these, 3C-like protease (3CLpro), papain-like protease (PLpro), and RNA-dependent RNA polymerase (RdRp) have been suggested to be targets for antiviral drug discovery (Totura and Bavari, 2019). In contrast to the structural and *nsp* proteins, amino acid conservation in accessory proteins is low, which makes them unsuitable drug targets.

A recent mass spectrometry analysis identified 332 putative human targets for therapeutical drugs (Gordon et al., 2020). Screening of over 60 potential leads resulted in two classes of molecules that reduced effectively viral infectivity. These molecules were protein biogenesis inhibitors (zotatifin, ternatin-4, and PS3061) and ligands of Sigma1 and Sigma2 receptors (haloperidol, PB28, PD-144418, and hydroxychloroquine) (Gordon et al., 2020). In addition, clemastine, cloperastine, siramesine, zotatifin, and progesterone were also identified as potential drugs in this study.

## Outlook

Much effort is currently ongoing to find a specific vaccine to protect populations against the threat of SARS-CoV-2. However, the development of a new vaccine is a long process that will come too late for hundreds of thousands of already infected people. Antiviral therapeutics such as remdesivir, lopinavir, ritonavir, favipiravir, or hydroxy-chloroquine, combined with immunotherapy, could work, and some of them are being used in the front line against the disease. However, new drugs that target specifically the new virus are needed to increase our weaponry in the fight of this pandemic emergency. To this end, small peptides containing the sequence of the ACE receptor directly involved in SARS-CoV-2 binding might be a promising alternative in the fight against this pandemic emergency. The detailed analysis of ACE-2 spike viral protein interactions carried out in this work suggests putative peptides that might fulfill this requirement, as well as certain ACE2 genetic variants that might be associated with a lower risk of infection. These molecules, combined with panviral strategies and drugs targeting other mechanisms of viral infection, could provide effective therapies for the management of the disease.

## Author Contributions

EC and IA wrote the article. IA designed and prepared all the figures. EC analyzed the polymorphisms and compiled data in Tables 1, 2. All authors contributed to the article and approved the submitted version.

## Conflict of Interest

The authors declare that the research was conducted in the absence of any commercial or financial relationships that could be construed as a potential conflict of interest.

## References

[B1] Adams M. J. Carstens E. B. (2012). Ratification vote on taxonomic proposals to the International Committee on Taxonomy of Viruses (2012). *Arch. Virol.* 157 1411–1422. 10.1007/s00705-012-1299-6 22481600 PMC7086667

[B2] Al-Tawfiq J. A. Momattin H. Dib J. Memish Z. A. (2014). Ribavirin and interferon therapy in patients infected with the Middle East respiratory syndrome coronavirus: an observational study. *Int. J. Infect. Dis.* 20 42–46. 10.1016/j.ijid.2013.12.003 24406736 PMC7110882

[B3] Anderson R. M. Fraser C. Ghani A. C. Donnelly C. A. Riley S. Ferguson N. M. (2004). Epidemiology, transmission dynamics and control of SARS: the 2002-2003 epidemic. *Philos. Trans. R. Soc. B Biol. Sci.* 359 1091–1105. 10.1098/rstb.2004.1490 15306395 PMC1693389

[B4] Assiri A. McGeer A. Perl T. M. Price C. S. Al Rabeeah A. A. Cummings D. A. T. (2013). Hospital outbreak of middle east respiratory syndrome coronavirus. *N. Engl. J. Med.* 369 407–416. 10.1056/NEJMoa1306742 23782161 PMC4029105

[B5] Babcock G. J. Esshaki D. J. Thomas W. D. Ambrosino D. M. (2004). Amino Acids 270 to 510 of the Severe Acute Respiratory Syndrome Coronavirus Spike Protein Are Required for Interaction with Receptor. *J. Virol.* 78 4552–4560. 10.1128/jvi.78.9.4552-4560.2004 15078936 PMC387703

[B6] Baron S. A. Devaux C. Colson P. Raoult D. Rolain J. M. (2020). Teicoplanin: an alternative drug for the treatment of COVID-19? *Int. J. Antimicrob. Agents* 55:105944. 10.1016/j.ijantimicag.2020.105944 32179150 PMC7102624

[B7] Batlle D. Wysocki J. Satchell K. (2020). Soluble angiotensin-converting enzyme 2: a potential approach for coronavirus infection therapy? *Clin. Sci.* 134 543–545. 10.1042/CS20200163 32167153

[B8] Belouzard S. Chu V. C. Whittaker G. R. (2009). Activation of the SARS coronavirus spike protein via sequential proteolytic cleavage at two distinct sites. *Proc. Natl. Acad. Sci. U.S.A.* 106 5871–5874. 19321428 10.1073/pnas.0809524106 PMC2660061

[B9] Bixler S. L. Bocan T. M. Wells J. Wetzel K. S. Van Tongeren S. A. Dong L. (2018). Efficacy of favipiravir (T-705) in nonhuman primates infected with Ebola virus or Marburg virus. *Antiviral Res.* 151 97–104. 10.1016/j.antiviral.2017.12.021 29289666

[B10] Bongini P. Trezza A. Bianchini M. Spiga O. Niccolai N. (2020). A possible strategy to fight COVID-19: interfering with spike glycoprotein trimerization. *Biochem. Biophys. Res. Commun.* 528 35–38. 10.1016/j.bbrc.2020.04.007 32451080 PMC7144664

[B11] Bosch B. J. van der Zee R. de Haan C. A. M. Rottier P. J. M. (2003). The coronavirus spike protein is a class I virus fusion protein:structural and functional characterization of the FusionCore Complex. *J. Ofvirol.* 77 8801–8811. 10.1128/jvi.77.16.8801-8811.2003 12885899 PMC167208

[B12] Bosch B. J. Martina B. E. E. Van Der Zee R. Lepault J. Haijema B. J. Versluis C. (2004). Severe acute respiratory syndrome coronavirus (SARS-CoV) infection inhibition using spike protein heptad repeat-derived peptides. *Proc. Natl. Acad. Sci. U.S.A.* 101 8455–8460. 10.1073/pnas.0400576101 15150417 PMC420415

[B13] Cai (2020). Correspondence sex difference and smoking predisposition smoking or vaping may increase the risk of a severe coronavirus infection. *Lancet* 2600 19–20.

[B14] Cao B. Wang Y. Wen D. Liu W. Wang J. Fan G. (2020). A trial of lopinavir-ritonavir in adults hospitalized with severe Covid-19. *N. Engl. J. Med.* 382 1787–1799. 10.1056/NEJMoa2001282 32187464 PMC7121492

[B15] Cao Y. Li L. Feng Z. Wan S. Huang P. Sun X. (2020). Comparative genetic analysis of the novel coronavirus (2019-nCoV/SARS-CoV-2) receptor ACE2 in different populations. *Cell Discov.* 6 4–7. 10.1038/s41421-020-0147-1 32133153 PMC7040011

[B16] Chan J. F. W. Yao Y. Yeung M. L. Deng W. Bao L. Jia L. (2015). Treatment with lopinavir/ritonavir or interferon-β1b improves outcome of MERSCoV infection in a nonhuman primate model of common marmoset. *J. Infect. Dis.* 212 1904–1913. 10.1093/infdis/jiv392 26198719 PMC7107395

[B17] Chu C. M. Cheng V. C. C. Hung I. F. N. Wong M. M. L. Chan K. H. Chan K. S. (2004). Role of lopinavir/ritonavir in the treatment of SARS: initial virological and clinical findings. *Thorax* 59 252–256. 10.1136/thorax.2003.012658 14985565 PMC1746980

[B18] Ciaglia E. Vecchione C. Puca A. A. (2020). COVID-19 infection and circulating ACE2 levels: protective role in women and children. *Front. Pediatr.* 8:206. 10.3389/fped.2020.00206 32391299 PMC7192005

[B19] Colson P. Rolain J. M. Raoult D. (2020). Chloroquine for the 2019 novel coronavirus SARS-CoV-2. *Int. J. Antimicrob. Agents* 55:105923. 10.1016/j.ijantimicag.2020.105923 32070753 PMC7134866

[B20] Coutard B. Valle C. de Lamballerie X. Canard B. Seidah N. G. Decroly E. (2020). The spike glycoprotein of the new coronavirus 2019-nCoV contains a furin-like cleavage site absent in CoV of the same clade. *Antiviral Res.* 176:104742. 10.1016/j.antiviral.2020.104742 32057769 PMC7114094

[B21] Crackower M. A. Sarao R. Oudit G. Y. Yagil C. Kozieradzki I. Scanga S. E. (2002). Angiotensin-converting enzyme 2 is an essential regulator of heart function. *Nature* 417 822–828. 10.1038/nature00786 12075344

[B22] Dahms S. O. Jiao G.-S. Than M. E. (2017). Structural Studies Revealed Active Site Distortions of Human Furin by a Small Molecule Inhibitor. *ACS Chem. Biol.* 12 1211–1216. 10.1021/acschembio.6b01110 28402100

[B23] De Wit E. Van Doremalen N. Falzarano D. Munster V. J. (2016). SARS and MERS: recent insights into emerging coronaviruses. *Nat. Rev. Microbiol.* 14 523–534. 10.1038/nrmicro.2016.81 27344959 PMC7097822

[B24] Devaux C. A. Rolain J.-M. Raoult D. (2020). ACE2 receptor polymorphism: susceptibility to SARS-CoV-2, hypertension, multi-organ failure, and COVID-19 disease outcome. *J. Microbiol. Immunol. Infect.* 53 425–435. 10.1016/j.jmii.2020.04.015 32414646 PMC7201239

[B25] Donoghue M. Hsieh F. Baronas E. Godbout K. Gosselin M. Stagliano N. (2000). UltraRapid Communication A Novel Angiotensin-Converting Enzyme – Related to Angiotensin 1-9. *Circ. Res.* 87 e1–e9. 10969042 10.1161/01.res.87.5.e1

[B26] Du L. He Y. Zhou Y. Liu S. Zheng B. J. Jiang S. (2009). The spike protein of SARS-CoV - A target for vaccine and therapeutic development. *Nat. Rev. Microbiol.* 7 226–236. 10.1038/nrmicro2090 19198616 PMC2750777

[B27] Eaaswarkhanth M. Al Madhoun A. Al-Mulla F. (2020). Could the D614G substitution in the SARS-CoV-2 spike (S) protein be associated with higher COVID-19 mortality? *Int. J. Infect. Dis.* 96 459–460. 10.1016/j.ijid.2020.05.071 32464271 PMC7247990

[B28] Elshabrawy H. A. Coughlin M. M. Baker S. C. Prabhakar B. S. (2012). Human Monoclonal Antibodies against Highly Conserved HR1 and HR2 Domains of the SARS-CoV Spike Protein Are More Broadly Neutralizing. *PLoS One* 7:e50366. 10.1371/journal.pone.0050366 23185609 PMC3503966

[B29] Feld J. J. Hoofnagle J. H. (2005). Mechanism of action of interferon and ribavirin in treatment of hepatitis C. *Nature* 436 967–972. 10.1038/nature04082 16107837

[B30] Fu B. Xu X. Wei H. (2020). Why tocilizumab could be an effective treatment for severe COVID-19? *J. Transl. Med.* 18 1–5. 10.1186/s12967-020-02339-3 32290839 PMC7154566

[B31] Furuta Y. Gowen B. B. Takahashi K. Shiraki K. Smee D. F. Barnard D. L. (2013). Favipiravir (T-705), a novel viral RNA polymerase inhibitor. *Antiviral Res.* 100 446–454. 10.1016/j.antiviral.2013.09.015 24084488 PMC3880838

[B32] Gallagher T. M. Buchmeier M. J. (2001). Coronavirus spike proteins in viral entry and pathogenesis. *Virology* 279 371–374. 10.1006/viro.2000.0757 11162792 PMC7133764

[B33] Gao J. Lu G. Qi J. Li Y. Wu Y. Deng Y. (2013). Structure of the Fusion core and inhibition of fusion by a heptad repeat peptide derived from the S protein of middle east respiratory syndrome coronavirus. *J. Virol.* 87 13134–13140. 10.1128/jvi.02433-13 24067982 PMC3838252

[B34] Gao J. Tian Z. Yang X. (2020). Breakthrough: Chloroquine phosphate has shown apparent efficacy in treatment of COVID-19 associated pneumonia in clinical studies. *Biosci. Trends* 14 72–73. 10.5582/BST.2020.01047 32074550

[B35] Gautret P. Lagier J.-C. Parola P. Hoang V. T. Meddeb L. Mailhe M. (2020). Hydroxychloroquine and azithromycin as a treatment of COVID-19: results of an open-label non-randomized clinical trial. *Int. J. Antimicrob. Agents* 2020:105949. 10.1016/j.ijantimicag.2020.105949 32205204 PMC7102549

[B36] Glowacka I. Bertram S. Muller M. A. Allen P. Soilleux E. Pfefferle S. (2011). Evidence that TMPRSS2 activates the severe acute respiratory syndrome coronavirus spike protein for membrane fusion and reduces viral control by the humoral immune response. *J. Virol.* 85 4122–4134. 10.1128/jvi.02232-10 21325420 PMC3126222

[B37] Goldhill D. H. Te Velthuis A. J. W. Fletcher R. A. Langat P. Zambon M. Lackenby A. (2018). The mechanism of resistance to favipiravir in influenza. *Proc. Natl. Acad. Sci. U.S.A.* 115 11613–11618. 10.1073/pnas.1811345115 30352857 PMC6233120

[B38] Gordon D. E. Jang G. M. Bouhaddou M. Xu J. Obernier K. Kris M. (2020). A SARS-CoV-2 protein interaction map reveals targets for drug repurposing. *Nature* 583 459–468. 10.1038/s41586-020-2286-9 32353859 PMC7431030

[B39] Grein J. Ohmagari N. Shin D. Diaz G. Asperges E. Castagna A. (2020). Compassionate use of remdesivir for patients with severe Covid-19. *N. Engl. J. Med.* 382 2327–2336. 10.1056/nejmoa2007016 32275812 PMC7169476

[B40] Guan W. J. Ni Z. Y. Hu Y. Liang W. H. Ou C. Q. He J. X. (2020). Clinical characteristics of coronavirus Disease 2019 in China. *N. Engl. J. Med.* 382 1708–1720. 10.1056/NEJMoa2002032 32109013 PMC7092819

[B41] Guo C. Li B. Ma H. Wang X. Cai P. Yu Q. (2020). Tocilizumab treatment in severe COVID-19 patients attenuates the inflammatory storm incited by monocyte centric immune interactions revealed by single-cell analysis. *bioRxiv* [Preprint]. 10.1101/2020.04.08.029769

[B42] Hoffmann M. Kleine-Weber H. Schroeder S. Krüger N. Herrler T. Erichsen S. (2020). SARS-CoV-2 Cell Entry Depends on ACE2 and TMPRSS2 and Is Blocked by a Clinically Proven Protease Inhibitor. *Cell* 181 271–280.e8. 10.1016/j.cell.2020.02.052 32142651 PMC7102627

[B43] Hu H. Li L. Kao R. Y. Kou B. Wang Z. Zhang L. (2005). Screening and Identification of Linear B-Cell Epitopes and Entry-Blocking Peptide of Severe Acute Respiratory Syndrome (SARS)-Associated Coronavirus Using Synthetic Overlapping Peptide Library. *J. Comb. Chem.* 7 648–656. 10.1021/cc0500607 16153058

[B44] Hussain M. Jabeen N. Raza F. Shabbir S. Baig A. A. Amanullah A. (2020). Structural variations in human ACE2 may influence its binding with SARS-CoV-2 spike protein. *J. Med. Virol.* 10.1002/jmv.25832 [Online ahead of print]. 32249956 PMC7228372

[B45] Izaguirre G. (2019). The Proteolytic Regulation of Virus Cell Entry by Furin and other Preprotein convertases. *Viruses* 11 837–856. 10.3390/v11090837 31505793 PMC6784293

[B46] Kawase M. Shirato K. van der Hoek L. Taguchi F. Matsuyama S. (2012). Simultaneous treatment of human bronchial epithelial cells with serine and Cysteine protease inhibitors prevents severe acute respiratory syndrome coronavirus entry. *J. Virol.* 86 6537–6545. 10.1128/jvi.00094-12 22496216 PMC3393535

[B47] Kim D. Lee J. Yang J. Kim J. W. Kim V. N. Chang H. (2020). The architecture of SARS-CoV-2 transcriptome resource the architecture of SARS-CoV-2 Transcriptome. *Cell*, 181 914–921.e10. 10.1016/j.cell.2020.04.011 32330414 PMC7179501

[B48] Kim U. J. Won E.-J. Kee S.-J. Jung S.-I. Jang H.-C. (2015). Combination therapy with lopinavir/ritonavir, ribavirin and interferon-alpha for Middle East respiratory syndrome: a case report. *Antivir. Ther.* 21 455–459. 10.3851/IMP3002 26492219

[B49] Kin N. Miszczak F. Lin W. Ar Gouilh M. Vabret A. Consortium E. (2015). Genomic analysis of 15 human coronaviruses OC43 (HCoV-OC43s) circulating in France from 2001 to 2013 reveals a high intra-specific diversity with new recombinant genotypes. *Viruses* 7 2358–2377. 10.3390/v7052358 26008694 PMC4452910

[B50] Kirchdoerfer R. N. Cottrell C. A. Wang N. Pallesen J. Yassine H. M. Turner H. L. (2016). Pre-fusion structure of a human coronavirus spike protein. *Nature* 531 118–121. 10.1038/nature17200 26935699 PMC4860016

[B51] Korber B. Fischer W. M. Gnanakaran S. Yoon H. Theiler J. Abfalterer W. (2020). Tracking changes in SARS-CoV-2 Spike: evidence that D614G increases infectivity of the COVID-19 virus. *Cell* 10.1016/j.cell.2020.06.043 [Online ahead of print]. 32697968 PMC7332439

[B52] Kruse R. L. (2020). Therapeutic strategies in an outbreak scenario to treat the novel coronavirus originating in Wuhan, China. *F1000Research* 9:72 10.12688/f1000research.22211.1 PMC7029759 32117569

[B53] Ksiazek T. G. Erdman D. Goldsmith C. S. Zaki S. R. Peret T. Emery S. (2003). A novel coronavirus associated with severe acute respiratory syndrome. *N. Engl. J. Med.* 348 1953–1966. 10.1056/NEJMoa030781 12690092

[B54] Kuiken T. Fouchier R. A. M. Schutten M. Rimmelzwaan G. F. Van Amerongen G. Van Riel D. (2003). Newly discovered coronavirus as the primary cause of severe acute respiratory syndrome. *Lancet* 362 263–270. 10.1016/S0140-6736(03)13967-0 12892955 PMC7112434

[B55] Lai M. M. C. Perlman S. Anderson L. J. (2007). “Coronaviridae,” in *Fields Virology*, eds Knipe D. M. Howley P. M. (Philadelphia, PA: Lippincott Williams & Wilkins), 1306–1335.

[B56] Lei C. Qian K. Li T. Zhang S. Fu W. Ding M. (2020). Neutralization of SARS-CoV-2 spike pseudotyped virus by recombinant ACE2-Ig. *Nat. Commun.* 11:2070. 10.1038/s41467-020-16048-4 32332765 PMC7265355

[B57] Li F. (2015). Receptor Recognition Mechanisms of Coronaviruses: a Decade of Structural Studies. *J. Virol.* 89 1954–1964. 10.1128/jvi.02615-14 25428871 PMC4338876

[B58] Li F. Berardi M. Li W. Farzan M. Dormitzer P. R. Harrison S. C. (2006). Conformational states of the severe acute respiratory syndrome coronavirus spike protein ectodomain. *J. Virol.* 80 6794–6800. 10.1128/jvi.02744-05 16809285 PMC1489032

[B59] Li F. Li W. Farzan M. Harrison S. C. (2005). Structural biology: structure of SARS coronavirus spike receptor-binding domain complexed with receptor. *Science* 309 1864–1868. 10.1126/science.1116480 16166518

[B60] Liu J. Cao R. Xu M. Wang X. Zhang H. Hu H. (2020). Hydroxychloroquine, a less toxic derivative of chloroquine, is effective in inhibiting SARS-CoV-2 infection in vitro. *Cell Discov.* 6 6–9. 10.1038/s41421-020-0156-0 32194981 PMC7078228

[B61] Liu S. Xiao G. Chen Y. He Y. Niu J. Escalante C. R. (2004). Mechanisms of disease Interaction between heptad repeat 1 and 2 regions in spike protein of SARS-associated coronavirus: implications for virus fusogenic mechanism and identification of fusion inhibitors. *Lancet* 363 938–947. 10.1016/S0140-6736(04)15788-7 15043961 PMC7140173

[B62] Lu L. Liu Q. Zhu Y. Chan K. H. Qin L. Li Y. (2014). Structure-based discovery of Middle East respiratory syndrome coronavirus fusion inhibitor. *Nat. Commun.* 5:3067. 10.1038/ncomms4067 24473083 PMC7091805

[B63] Mehra M. R. Desai S. S. Kuy S. Henry T. D. Patel A. N. (2020a). Cardiovascular disease, drug therapy, and mortality in Covid-19. *N. Engl. J. Med.* 382:e102. 10.1056/NEJMoa2007621 32356626 PMC7206931

[B64] Mehra M. R. Desai S. S. Ruschitzka F. Patel A. N. (2020b). Articles Hydroxychloroquine or chloroquine with or without a macrolide for treatment of COVID-19: a multinational registry analysis. *Lancet* 6736 1–10. 10.1016/S0140-6736(20)31180-6 PMC7255293 32450107

[B65] Meyerholz D. K. Lambertz A. M. Mccray P. B. (2016). Dipeptidyl Peptidase 4 Distribution in the Human Respiratory Tract Implications for the Middle East Respiratory Syndrome. *Am. J. Pathol.* 186 78–86. 10.1016/j.ajpath.2015.09.014 26597880 PMC4715219

[B66] Millet J. K. Whittaker G. R. (2014). Host cell entry of Middle East respiratory syndrome coronavirus after two-step, furin-mediated activation of the spike protein. *Proc. Natl. Acad. Sci. U.S.A.* 111 15214–15219. 10.1073/pnas.1407087111 25288733 PMC4210292

[B67] Millet J. K. Whittaker G. R. (2015). Host cell proteases: critical determinants of coronavirus tropism and pathogenesis. *Virus Res.* 202 120–134. 10.1016/j.virusres.2014.11.021 25445340 PMC4465284

[B68] Pant S. Singh M. Ravichandiran V. Murty U. S. N. Srivastava H. K. (2020). Peptide-like and small-molecule inhibitors against Covid-19. *J. Biomol. Struct. Dyn.* 10.1080/07391102.2020.1757510 [Online ahead of print]. 32306822 PMC7212534

[B69] Pettersen E. F. Goddard T. D. Huang C. C. Couch G. S. Greenblatt D. M. Meng E. C. (2004). UCSF Chimera?A visualization system for exploratory research and analysis. *J. Comput. Chem.* 25 1605–1612. 10.1002/jcc.20084 15264254

[B70] Phelan J. Deelder W. Ward D. Campino S. Hibberd M. L. Clark T. G. (2020). Controlling the SARS-CoV-2 outbreak, insights from large scale whole genome sequences generated across the world. *bioRxiv.* [Preprint] 10.1101/2020.04.28.066977

[B71] Prabakaran P. Gan J. Feng Y. Zhu Z. Choudhry V. Xiao X. (2006). Structure of severe acute respiratory syndrome coronavirus receptor-binding domain complexed with neutralizing antibody ^∗^. *J. Biol. Chem.* 281 15829–15836. 10.1074/jbc.M600697200 16597622 PMC8099238

[B72] Qiu M. Shi Y. Guo Z. Chen Z. He R. Chen R. (2005). Antibody responses to individual proteins of SARS coronavirus and their neutralization activities. *Microbes Infect.* 7 882–889. 10.1016/j.micinf.2005.02.006 15878679 PMC7110836

[B73] Qureshi A. Tantray V. G. Kirmani A. R. Ahangar A. G. (2018). A review on current status of antiviral siRNA. *Rev. Med. Virol.* 28 1–11. 10.1002/rmv.1976 29656441 PMC7169094

[B74] Reinke L. M. Spiegel M. Plegge T. Hartleib A. Nehlmeier I. Gierer S. (2017). Different residues in the SARS-CoV spike protein determine cleavage and activation by the host cell protease TMPRSS2. *PLoS One* 12:e0179177. 10.1371/journal.pone.0179177 28636671 PMC5479546

[B75] Rota P. A. Oberste M. A. Monroe S. Nix W. A. Campagnoli R. Icenogle J. P. (2003). Characterization of a novel coronavirus associated with severe acute respiratory syndrome. *Science* 300 1394–1399. 10.1126/science.1085952 12730500

[B76] Seidah N. G. Prat A. (2012). The biology and therapeutic targeting of the proprotein convertases. *Nat. Rev. Drug Discov.* 11 367–383. 10.1038/nrd3699 22679642

[B77] Shang J. Wan Y. Liu C. Yount B. Gully K. Yang Y. (2020a). Structure of mouse coronavirus spike protein complexed with receptor reveals mechanism for viral entry. *PLoS Pathog.* 16:e8392. 10.1371/journal.ppat.1008392 32150576 PMC7082060

[B78] Shang J. Ye G. Shi K. Wan Y. Luo C. Aihara H. (2020b). Structural basis of receptor recognition by SARS-CoV-2. *Nature* 581 221–224. 10.1038/s41586-020-2179-y 32225175 PMC7328981

[B79] Sheahan T. P. Sims A. C. Leist S. R. Schäfer A. Won J. Brown A. J. (2020). Comparative therapeutic efficacy of remdesivir and combination lopinavir, ritonavir, and interferon beta against MERS-CoV. *Nat. Commun.* 11:222. 10.1038/s41467-019-13940-6 31924756 PMC6954302

[B80] Shin Y. W. Chang K.-H. Hong G.-W. Yeo S.-G. Jee Y. Kim J.-H. (2019). Selection of Vaccinia Virus-Neutralizing Antibody from a Phage-Display Human-Antibody Library. *J. Microbiol. Biotechnol.* 29 651–657. 10.4014/jmb.1812.12024 30856707

[B81] Siegel D. Hui H. C. Doerffler E. Clarke M. O. Chun K. Zhang L. (2017). Discovery and Synthesis of a Phosphoramidate Prodrug of a Pyrrolo[2,1-f][triazin-4-amino] Adenine C-Nucleoside (GS-5734) for the treatment of Ebola and emerging viruses. *J. Med. Chem.* 60 1648–1661. 10.1021/acs.jmedchem.6b01594 28124907

[B82] Simmons G. Bertram S. Glowacka I. Steffen I. Chaipan C. Agudelo J. (2011). Different host cell proteases activate the SARS-coronavirus spike-protein for cell-cell and virus-cell fusion. *Virology* 413 265–274. 10.1016/j.virol.2011.02.020 21435673 PMC3086175

[B83] Song Z. Xu Y. Bao L. Zhang L. Yu P. Qu Y. (2019). From SARS to MERS, thrusting coronaviruses into the spotlight. *Viruses* 11:E59. 10.3390/v11010059 30646565 PMC6357155

[B84] Struck A. W. Axmann M. Pfefferle S. Drosten C. Meyer B. (2012). A hexapeptide of the receptor-binding domain of SARS corona virus spike protein blocks viral entry into host cells via the human receptor ACE2. *Antiviral Res.* 94 288–296. 10.1016/j.antiviral.2011.12.012 22265858 PMC7114193

[B85] Su S. Wong G. Shi W. Liu J. Lai A. C. K. Zhou J. (2016). Epidemiology, genetic recombination, and pathogenesis of coronaviruses. *Trends Microbiol.* 24 490–502. 10.1016/j.tim.2016.03.003 27012512 PMC7125511

[B86] Sui J. Li W. Murakami A. Tamin A. Matthews L. J. Wong S. K. (2004). Potent neutralization of severe acute respiratory syndrome (SARS) coronavirus by a human mAb to S1 protein that blocks receptor association. *Proc. Natl. Acad. Sci. U.S.A.* 101 2536–2541. 10.1073/pnas.0307140101 14983044 PMC356985

[B87] Sun Y. Zhang H. Shi J. Zhang Z. Gong R. (2017). Identification of a novel inhibitor against middle east respiratory syndrome coronavirus. *Viruses* 9 1–12. 10.3390/v9090255 28906430 PMC5618021

[B88] Tchesnokov E. P. Feng J. Y. Porter D. P. Götte M. (2019). Mechanism of inhibition of ebola virus RNA-dependent RNA polymerase by remdesivir. *Viruses* 11 1–16. 10.3390/v11040326 30987343 PMC6520719

[B89] Te H. S. Randall G. Jensen D. M. (2007). Mechanism of action of ribavirin in the treatment of chronic hepatitis C. *Gastroenterol. Hepatol.* 3 218–225. PMC3099343 21960835

[B90] Ter Meulen J. Bakker A. B. H. Van Den Brink E. N. Weverling G. J. Martina B. E. E. Haagmans B. L. (2004). Human monoclonal antibody as prophylaxis for SARS coronavirus infection in ferrets. *Lancet* 363 2139–2141. 10.1016/S0140-6736(04)16506-9 15220038 PMC7112500

[B91] Thi E. P. Mire C. E. Lee A. C. H. Geisbert J. B. Zhou J. Z. Agans N. (2015). Infected Nonhuman Primates. *Nature* 521 362–365. 10.1038/nature14442.Lipid 25901685 PMC4467030

[B92] Tian X. Li C. Huang A. Xia S. Lu S. Shi Z. (2020). Potent binding of 2019 novel coronavirus spike protein by a SARS coronavirus-specific human monoclonal antibody. *Emerg. Microbes Infect.* 9 382–385. 10.1080/22221751.2020.1729069 32065055 PMC7048180

[B93] Totura A. L. Bavari S. (2019). Broad-spectrum coronavirus antiviral drug discovery. *Expert Opin. Drug Discov.* 14 397–412. 10.1080/17460441.2019.1581171 30849247 PMC7103675

[B94] Towler P. Staker B. Prasad S. G. Menon S. Tang J. Parsons T. (2004). ACE2 X-Ray structures reveal a large hinge-bending motion important for inhibitor binding and catalysis. *J. Biol. Chem.* 279 17996–18007. 10.1074/jbc.M311191200 14754895 PMC7980034

[B95] van den Brink E. N. ter Meulen J. Cox F. Jongeneelen M. A. C. Thijsse A. Throsby M. (2005). Molecular and biological characterization of human monoclonal antibodies binding to the spike and nucleocapsid proteins of severe acute respiratory syndrome coronavirus. *J. Virol.* 79 1635–1644. 10.1128/jvi.79.3.1635-1644.2005 15650189 PMC544131

[B96] Walker L. M. Burton D. R. (2018). Passive immunotherapy of viral. *Nat. Publ. Gr.* 18 297–308. 10.1038/nri.2017.148 29379211 PMC5918154

[B97] Walls A. C. Park Y. J. Tortorici M. A. Wall A. McGuire A. T. Veesler D. (2020). Structure, Function, and Antigenicity of the SARS-CoV-2 Spike Glycoprotein. *Cell* 181 281–292.e6. 10.1016/j.cell.2020.02.058 32155444 PMC7102599

[B98] Walls A. C. Tortorici M. A. Bosch B. J. Frenz B. Rottier P. J. M. DiMaio F. (2016). Cryo-electron microscopy structure of a coronavirus spike glycoprotein trimer. *Nature* 531 114–117. 10.1038/nature16988 26855426 PMC5018210

[B99] Walls A. C. Xiong X. Park Y. J. Tortorici M. A. Snijder J. Quispe J. (2019). Unexpected Receptor Functional Mimicry Elucidates Activation of Coronavirus Fusion. *Cell* 176 1026–1039.e15. 10.1016/j.cell.2018.12.028 30712865 PMC6751136

[B100] Wang M. Cao R. Zhang L. Yang X. Liu J. Xu M. (2020). Remdesivir and chloroquine effectively inhibit the recently emerged novel coronavirus (2019-nCoV) in vitro. *Cell Res.* 30 269–271. 10.1038/s41422-020-0282-0 32020029 PMC7054408

[B101] Wang Q. Zhang Y. Wu L. Niu S. Song C. Zhang Z. (2020). Structural and Functional Basis of SARS-CoV-2 Entry by Using Human ACE2. *Cell* 181 894–904.e9. 10.1016/j.cell.2020.03.045 32275855 PMC7144619

[B102] Wang Y. Zhang D. Du P. G. Du P. R. Zhao P. J. Jin P. Y. (2020). Articles Remdesivir in adults with severe COVID-19: a randomised, double-blind, placebo-controlled, multicentre trial. *Lancet* 6736 1–10. 10.1016/S0140-6736(20)31022-9 PMC7190303 32423584

[B103] Watanabe Y. Allen J. D. Wrapp D. McLellan J. S. Crispin M. (2020). Site-specific glycan analysis of the SARS-CoV-2 spike. *Science* 369 330–333. 10.1126/science.abb9983 32366695 PMC7199903

[B104] Wrapp D. Wang N. Corbett K. S. Goldsmith J. A. Hsieh C. L. Abiona O. (2020). Cryo-EM structure of the 2019-nCoV spike in the prefusion conformation. *Science* 367 1260–1263. 10.1126/science.aax0902 32075877 PMC7164637

[B105] Wu C. Y. Jan J. T. Ma S. H. Kuo C. J. Juan H. F. Cheng Y. S. E. (2004). Small molecules targeting severe acute respiratory syndrome human coronavirus. *Proc. Natl. Acad. Sci. U.S.A.* 101 10012–10017. 10.1073/pnas.0403596101 15226499 PMC454157

[B106] Wysocki J. Ye M. Rodriguez E. González-Pacheco F. R. Barrios C. Evora K. (2010). Targeting the degradation of angiotensin II with recombinant angiotensin-converting enzyme 2: prevention of angiotensin II-dependent hypertension. *Hypertension* 55 90–98. 10.1161/HYPERTENSIONAHA.109.138420 19948988 PMC2827767

[B107] Xia S. Liu M. Wang C. Xu W. Lan Q. Feng S. (2020a). Inhibition of SARS-CoV-2 (previously 2019-nCoV) infection by a highly potent pan-coronavirus fusion inhibitor targeting its spike protein that harbors a high capacity to mediate membrane fusion. *Cell Res.* 30 343–355. 10.1038/s41422-020-0305-x 32231345 PMC7104723

[B108] Xia S. Zhu Y. Liu M. Lan Q. Xu W. Wu Y. (2020b). Fusion mechanism of 2019-nCoV and fusion inhibitors targeting HR1 domain in spike protein. *Cell. Mol. Immunol.* 17 765–767. 10.1038/s41423-020-0374-2 32047258 PMC7075278

[B109] Xia S. Xu W. Wang Q. Wang C. Hua C. Li W. (2018). Peptide-based membrane fusion inhibitors targeting HCOV-229E spike protein HR1 and HR2 domains. *Int. J. Mol. Sci.* 19 8–11. 10.3390/ijms19020487 29415501 PMC5855709

[B110] Xia S. Yan L. Xu W. Agrawal A. S. Algaissi A. Tseng C.-T. K. (2019). A pan-coronavirus fusion inhibitor targeting the HR1 domain of human coronavirus spike. *Sci. Adv.* 5:eaav4580. 10.1126/sciadv.aav4580 30989115 PMC6457931

[B111] Yan R. Zhang Y. Li Y. Xia L. Guo Y. Zhou Q. (2020). Structural basis for the recognition of SARS-CoV-2 by full-length human ACE2. *Science* 367 1444–1448. 10.1126/science.abb2762 32132184 PMC7164635

[B112] Yang T.-J. Chang Y.-C. Ko T.-P. Draczkowski P. Chien Y.-C. Chang Y.-C. (2020). Cryo-EM analysis of a feline coronavirus spike protein reveals a unique structure and camouflaging glycans. *Proc. Natl. Acad. Sci. U.S.A.* 117 1438–1446. 10.1073/pnas.1908898117 31900356 PMC6983407

[B113] Yuan K. Yi L. Chen J. Qu X. Qing T. Rao X. (2004). Suppression of SARS-CoV entry by peptides corresponding to heptad regions on spike glycoprotein. *Biochem. Biophys. Res. Commun.* 319 746–752. 10.1016/j.bbrc.2004.05.046 15184046 PMC7111000

[B114] Yuan M. Wu N. C. Zhu X. Lee C.-C. D. So R. T. Y. Lv H. (2020). A highly conserved cryptic epitope in the receptor binding domains of SARS-CoV-2 and SARS-CoV. *Science* 368 630–633. 10.1126/science.abb7269 32245784 PMC7164391

[B115] Zhang G. Pomplun S. Loftis A. R. Loas A. Pentelute B. L. (2020). The first-in-class peptide binder to the SARS-CoV-2 spike protein Affiliations: Massachusetts Institute of Technology, Department of Chemistry, 77 Massachusetts Avenue, Cambridge, MA 02139, USA. Extramural Member, Koch Institute of Integrative Can. *bioRxiv* [Preprint]. 10.1101/2020.03.19.999318

[B116] Zhang H. Wada J. Hida K. Tsuchiyama Y. Hiragushi K. Shikata K. (2001). Collectrin, a collecting duct-specific Transmembrane Glycoprotein, is a novel homolog of ACE2 and is developmentally regulated in embryonic kidneys. *J. Biol. Chem.* 276 17132–17139. 10.1074/jbc.M006723200 11278314

[B117] Zheng B. J. Guan Y. He M. L. Sun H. Du L. Zheng Y. (2005). Synthetic peptides outside the spike protein heptad repeat regions as potent inhibitors of SARS-associated coronavirus. *Antivir. Ther.* 10 393–403. 15918330

[B118] Zhou Y. Vedantham P. Lu K. Agudelo J. Carrion R. Nunneley J. W. (2015). Protease inhibitors targeting coronavirus and filovirus entry. *Antiviral Res.* 116 76–84. 10.1016/j.antiviral.2015.01.011 25666761 PMC4774534

[B119] Zhu Z. Chakraborti S. He Y. Roberts A. Sheahan T. Xiao D. (2007). Potent cross-reactive neutralization of SARS coronavirus isolates by human monoclonal antibodies. *Proc. Natl. Acad. Sci. U.S.A.* 104 12123–12128. 10.1073/pnas.0701000104 17620608 PMC1924550

