# Supplementary material for: Drug Weaponry to Fight Against SARS-CoV-2
Source: Front Mol Biosci. 2020 Aug 25;7:204. doi: 10.3389/fmolb.2020.00204 (PMC7477106; doi:10.3389/fmolb.2020.00204)
Supplement: Supplementary file 1 [file Image_1.pdf]

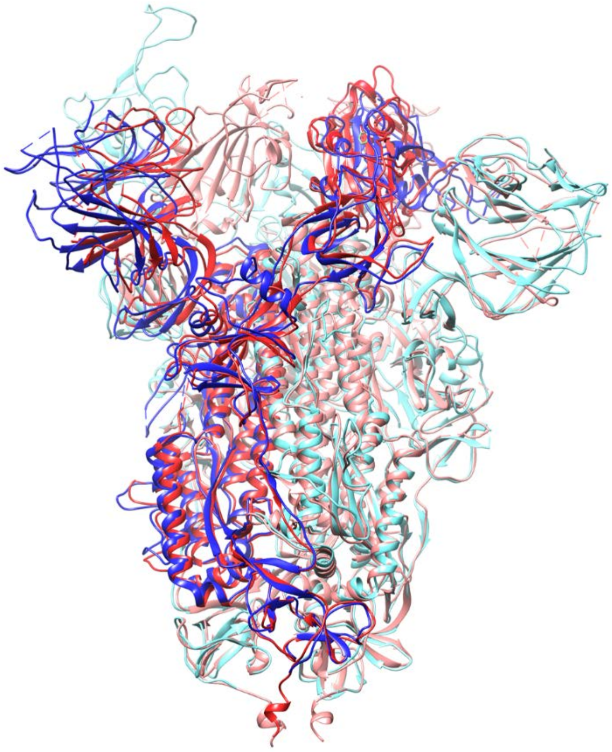

Supp. Fig 1**. Structural comparison of SARS-CoV and SARS-CoV-2 spike proteins**. Trimeric conformation of the spike S1 protein of SARS-CoV (cyan PDB id:6crz) (Kirchdoerfer et al., 2018) and SARS-CoV-2 (pink PDB id:6vsb) (Wrapp et al., 2020) in the prefusion conformation. A monomer of both SARS-CoV and SARS-CoV-2 spike proteins are highlighted in blue and red, respectively.

A

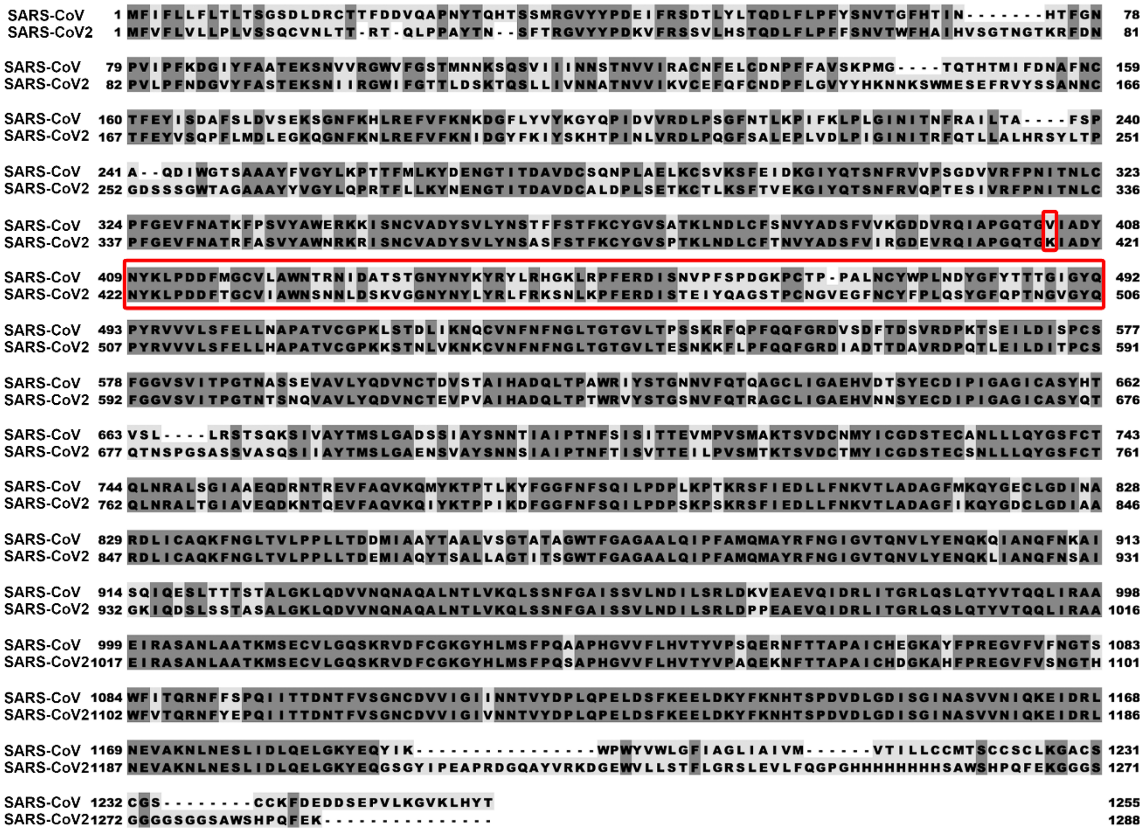

B

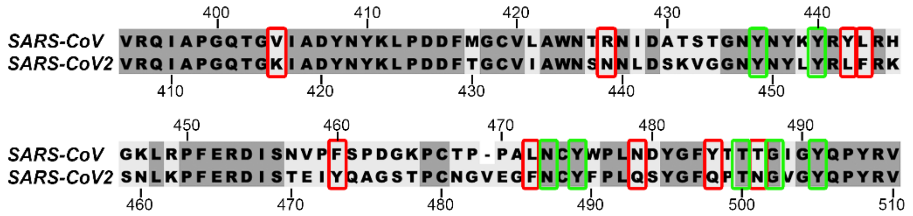

Supp. Fig. 2**. Sequence alignment of SARS-CoV and SARS-CoV-2 spike proteins**. Sequences of the spike S1 proteins of SARS-CoV and SARS-CoV-2 were aligned using T-coffee (Notredame et al., 2000) and illustrated using Jalview (Waterhouse et al. 2009). Sequence identity is expressed in dark grey colour. B, RBD (Receptor Binding Domain) residues of the spike protein involved in the interaction with the ACE-2 receptor are highlighted. Conserved residues in both proteins are marked with green boxes whereas those amino acids that are different are marked in red.

Supp. Fig. 3. **Amino acids involved in the interaction between the ACE-2 receptor and the RBD of the spike proteins of SARS-CoV and SARS-CoV-2 viruses.**

Contacts between ACE-2 and SARS-CoV according to Li et al., (2005) (central column) and SARS-CoV-2 (right column) (Yan et al., 2020). Residues conserved between SARS-CoV and SARS-CoV-2 are depicted in green and changes are coloured in red.

ACE-2	SARS-CoV	SARS-CoV-2
Q^{24}	N^{473}	N^{488}
T^{27}	Y^{475}	Y^{489}
D^{30}	V^{404}	K^{417}
K^{31}	Y^{475}, Y^{442}	Y^{489}, L^{454}
H^{34}	Y^{440}, N^{479}	L^{454}, Q^{493}
E^{37}	Y^{491}	Y^{505}
D^{38}	Y^{436}	Y^{449}
Y^{41}	Y^{484}, T^{486}, T^{487}	Q^{498}, T^{500}, N^{501}
Q^{42}	Y^{436}, Y^{484}	Y^{449}, Q^{498}
L^{45}	Y^{484}	Q^{498}
L^{79}	L^{472}	F^{484}
M^{82}	L^{472}	F^{484}
Y^{83}	N^{473}, Y^{475}	N^{487}, Y^{489}
N^{90}	T^{402}	T^{415}
Q^{325}	R^{426}	N^{439}
E^{329}	R^{426}	N^{439}
N^{330}	T^{486}	N^{501}
K^{353}	G^{488}, T^{487}, Y^{491}	G^{502}, N^{501}, Y^{505}
G^{354}	Y^{491}, G^{488}	Y^{505}, G^{502}

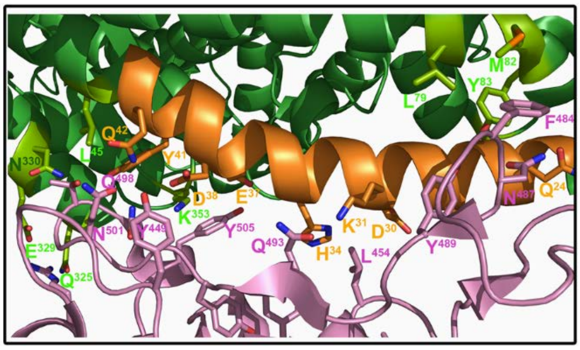

<u>Supp. Fig. 4.</u> **Amino acid residues of SARS-CoV-2 S1-viral spike protein involved in the interaction with the ACE-2 receptor**. The α1-helix of ACE-2 receptor is coloured in orange. A peptide containing these 23 residues (IEEQAKTFLDKFNHEAEDLFYQS) has been proposed to block the interaction between the virus and the receptor (Zhang et al., 2020).
